# Influence of pH value and salts on the adsorption of lysozyme in mixed‐mode chromatography

**DOI:** 10.1002/elsc.202100058

**Published:** 2021-11-08

**Authors:** Jannette Kreusser, Fabian Jirasek, Hans Hasse

**Affiliations:** ^1^ Laboratory of Engineering Thermodynamics (LTD) TU Kaiserslautern Kaiserslautern Germany

**Keywords:** adsorption isotherms, chromatography, modeling, pH, protein

## Abstract

Mixed‐mode chromatography (MMC) is an interesting technique for challenging protein separation processes which typically combines adsorption mechanisms of ion exchange (IEC) and hydrophobic interaction chromatography (HIC). Adsorption equilibria in MMC depend on multiple parameters but systematic studies on their influence are scarce. In the present work, the influence of the pH value and ionic strengths up to 3000 mM of four technically relevant salts (sodium chloride, sodium sulfate, ammonium chloride, and ammonium sulfate) on the lysozyme adsorption on the mixed‐mode resin Toyopearl MX‐Trp‐650M was studied systematically at 25℃. Equilibrium adsorption isotherms at pH 5.0 and 6.0 were measured and compared to experimental data at pH 7.0 from previous work. For all pH values, an exponential decay of the lysozyme loading with increasing ionic strength was observed. The influence of the pH value was found to depend significantly on the ionic strength with the strongest influence at low ionic strengths where increasing pH values lead to decreasing lysozyme loadings. Furthermore, a mathematical model that describes the influence of salts and the pH value on the adsorption of lysozyme in MMC is presented. The model enables predicting adsorption isotherms of lysozyme on Toyopearl MX‐Trp‐650M for a broad range of technically relevant conditions.

**Abbreviations**
HIC: hydrophobic interaction chromatography
HTS: high‐throughput screenings
IEC: ion exchange chromatography
IEP: isoelectric point
Lys: lysozyme
MMC: mixed‐mode chromatography

**Greek symbols**
β
: lumped fitting parameter
γ
: lumped fitting parameter

## 1 INTRODUCTION

Chromatographic purification techniques play an important role in biotechnological downstream processing [1, 2]. Mixed‐mode chromatography (MMC) has attracted much interest in recent years [3, 4], in particular for challenging protein separation processes as MMC combines at least two interaction modes, which can act individually or mutually. The most commonly used MMC resins are combinations of ion exchange chromatography (IEC) and hydrophobic interaction chromatography (HIC) resins [5]. At low ionic strengths, the ion exchange interactions between the protein molecules and the MMC resin are dominant, i.e., the MMC resin becomes similar to an IEC resin. The addition of salts not only weakens ionic interactions by shielding the surface charges on protein molecules and resin, but also enhances hydrophobic interactions [6]. Hence, at high ionic strengths, hydrophobic interactions become more important, i.e., the MMC resin behaves more like a HIC resin if sufficiently large amounts of salts are added. Mixed‐mode resins provide many advantages compared to traditional single‐mode resins: they can offer high binding capacities for proteins, in particular compared to single‐mode HIC resins [7], and improved selectivities due to the multitude of interaction mechanisms [8, 9]. Furthermore, a higher salt tolerance compared to IEC resins [10] usually enables wider operating ranges for MMC processes, which may even allow direct processing of high conductivity feedstocks [11]. Moreover, the simultaneous operation of different interaction modes can enable capturing and purification of target molecules from complex solutions in a single process step [12, 13].

In general, chromatographic processes for the purification of proteins build on complex adsorption mechanisms and there is still a lack of understanding these mechanisms in many cases. Therefore, conceptual process design of protein chromatography is often based on experimental high‐throughput screenings (HTS) in practice [14], which are less targeted and uneconomical. The higher complexity of MMC processes compared to conventional single‐mode chromatographic methods additionally hampers finding and selecting suitable or even optimized process conditions, as there are many parameters that have to be considered, such as pH value, ionic strength, and type of salt. To date, systematic experimental studies to describe the adsorption equilibria of proteins in MMC for a broad range of process parameters are still rare. Studies in the literature are mostly restricted to covering the influence of the pH value in salt‐free solutions or solutions containing sodium chloride only, e.g., [15, 16, 17]. In the present work, we fill this gap and provide a comprehensive study of the influence of multiple process parameters on the protein adsorption on a MMC resin; the MMC resin that is thereby considered combines IEC and HIC ligands.

**PRACTICAL APPLICATION**

In the present work, we provide a comprehensive experimental study of the influence of four different salts, the ionic strength, and the pH value on the adsorption of lysozyme on a mixed‐mode resin. We furthermore present a mathematical model for describing the influence of all parameters on the adsorption in the complete technically relevant ranges. The model allows the prediction of adsorption isotherms also for conditions not included in the training set. Our model approach is thereby generic and can be transferred to other systems (proteins, resins, salts) in a straightforward manner. It is therefore a valuable tool for applications in the simulation, the conceptual design, and the optimization of separation processes of proteins with mixed‐mode chromatography in general.

In single‐mode IEC, protein adsorption is based on attractive ionic interactions between the protein molecules and the resin, mainly due to opposite surface charges of the protein and the resin [18, 19, 20]. The net charge of the protein is thereby, in combination with the employed type of IEC resin, a suitable indicator for the adsorption of the protein. The net charge of a protein, in turn, strongly depends on the pH value: at pH values above its isoelectric point (IEP), a protein carries a negative net charge and favorably adsorbs on an anion exchange resin, whereas at pH values below its IEP, the protein carries a positive net charge and favorably adsorbs on a cation exchange resin. The elution of proteins in IEC is typically controlled by either shifting the proteins’ net charge by altering the pH value, or by increasing the ionic strength as ions present in the solution weaken the ionic interactions by shielding the protein molecules and the resin. Hence, pH value and ionic strength are both important process parameters to control adsorption and elution in IEC [1, 21].

In single‐mode HIC, by contrast, the adsorption of proteins is based on hydrophobic interactions between protein and resin. These hydrophobic interactions are especially favored by the presence of kosmotropic salts, as defined by the Hofmeister series [22], which affect the hydrate shell of protein molecules and HIC ligands and lead to increased adsorption by salting‐out effects [6, 23, 24]. However, kosmotropic salts do not always induce a stronger adsorption of proteins on HIC resins than chaotropic salts, i.e., protein adsorption cannot always be explained by the Hofmeister series. It is, for example, well‐known that lysozyme follows the Hofmeister series only at high ionic strengths and pH values, while at low ionic strengths and pH values, chaotropic salts are found to lead to a stronger adsorption of lysozyme on HIC resins [25, 26, 27]. Usually, protein adsorption in HIC is carried out at high ionic strengths of kosmotropic salts, while elution is controlled by reducing the ionic strength. As a consequence, ionic strength and type of salt present in solution are the predominant process parameters to control adsorption in HIC [28, 29].

Protein adsorption on MMC resins that combine IEC and HIC ligands is obviously more complex as process parameters affecting both single‐mode adsorption mechanisms have to be considered. Furthermore, single parameters can have opposing effects on the underlying mechanisms, i.e., favoring adsorption on IEC sites while weakening adsorption on HIC sites (e.g., low ionic strength), and vice versa. Hence, there are many ways to control adsorption and elution in MMC processes; most commonly, changing ionic strength or the pH value are considered, whereby pH‐gradient elution enables superior chromatographic resolution [9].

As a consequence of the insufficient experimental examination of adsorption equilibria of proteins in MMC to date, also suitable mathematical models for describing these adsorption equilibria are scarce. There are only few examples, which all have limited scope: the adsorption isotherm models of Ghose et al. [30] and Gao et al. [31] are based on a modified Langmuir equation and consider the effect of the pH value by including the concentration of hydrogen ions. However, only low ionic strengths up to 500 mM are considered in these approaches. The model of Nfor et al. [32], which is based on Mollerup's generalized thermodynamic framework for protein adsorption in IEC and HIC [2, 33, 34, 35], was applied for describing the influence of only sodium chloride at different pH values. To the best of our knowledge, no model considering the influence of the pH value and a wide range of ionic strengths for multiple salts on the adsorption of proteins on MMC resins has been reported yet.

In a recent study, we have developed a mathematical model for describing the influence of the ionic strength of four important salts (sodium chloride, sodium sulfate, ammonium chloride, and ammonium sulfate) on the lysozyme loading of a mixed‐mode resin at a constant pH value of 7.0 [36]. This model covers the entire range of technically relevant ionic strengths of the studied salts and follows the approach introduced in earlier work of our group for describing adsorption equilibria in HIC [37, 38, 39, 40], which has already been successfully applied for predicting elution profiles of proteins for the conceptual process design of HIC processes [41]. However, our current model [36] is not capable of describing the influence of the pH value; this gap is filled in the present work.

In the present work, we have systematically studied the influence of the pH value and the ionic strength of different salts on the adsorption of the model protein lysozyme on the mixed‐mode resin Toyopearl MX‐Trp‐650M in equilibrium adsorption experiments. The studied salts were sodium chloride, sodium sulfate, ammonium chloride, and ammonium sulfate and the ionic strength was varied between 0 and 3000 mM. All experiments were carried out at 25℃. As pH values, pH 5.0 and 6.0 were studied and the results were compared to those from our previous work at pH 7.0 [36]. Furthermore, the mathematical model from [36] that describes the adsorption of lysozyme on a mixed‐mode resin at a constant pH value was extended to also describe the influence of the pH value in the present work. The predictive capacity of the model was tested based on several adsorption isotherms at pH 8.0 that were additionally measured in the present work but not used for developing and fitting the model. The final model enables the prediction of equilibrium adsorption isotherms of lysozyme on Toyopearl MX‐Trp‐650M at 25℃ and any pH value in the range between approx. 5.0 and 8.0, and ionic strengths up to 3000 mM of the four studied salts, which basically covers the entire technically relevant range of these parameters. The presented approach should also be transferable to other systems (proteins, MMC resins, salts) in a straightforward manner and therefore constitutes a valuable tool for the conceptual design of MMC processes for protein separations in general.

## 2 MATERIALS AND METHODS

### 2.1 Materials

All materials used in the present work were the same as used in Kreusser et al. [36]. Hen egg white lysozyme (Lys, *M *= 14.3 kDa) with a purity over 90% was obtained from Sigma‐Aldrich and the mixed‐mode resin Toyopearl MX‐Trp‐650M, a methacrylic polymer‐based resin with a tryptophan ligand and an ion exchange capacity of 0.08–0.15 eq/L as specified by the supplier [42], was obtained from Tosoh Bioscience. The salts for the preparation of the buffers, namely, monosodium phosphate (NaH_2_PO_4_·2H_2_O) and disodium phosphate (Na_2_HPO_4_·2H_2_O), and the salts that were studied, namely, sodium chloride (NaCl), sodium sulfate (Na_2_SO_4_), ammonium chloride (NH_4_Cl), and ammonium sulfate ((NH_4_)_2_SO_4_), were of analytical grade. All salts were obtained from Carl Roth, except for ammonium chloride which was obtained from Bernd Kraft. For the adjustment of the pH value of the buffer and salt solutions, 1 N sodium hydroxide (NaOH) and 1 N hydrochloric acid (HCl) were used, which were obtained from Carl Roth. The high‐purity water used as solvent was produced with a Milli‐Q machine from Merck Millipore.

### 2.2 Experimental procedure and correlation of individual adsorption isotherms

Batch adsorption experiments were carried out on a fully automated liquid handling station Freedom EVO 200 from Tecan as described in detail in Kreusser et al. [36]. A 25 mM sodium phosphate buffer was prepared for each studied pH value. The pH value of the buffers was adjusted to 5.0, 6.0, or 8.0, respectively, with 1 N sodium hydroxide or 1 N hydrochloric acid. Preliminary lysozyme solutions and additional solutions of one of the studied salts (NaCl, Na_2_SO_4_, NH_4_Cl, or (NH_4_)_2_SO_4_) were prepared gravimetrically by dissolving lysozyme or the salts, respectively, in the corresponding 25 mM sodium phosphate buffer with the desired pH value. After dissolving the salts, the pH values of the salt solutions were revised and, if necessary, adjusted to pH 5.0, 6.0, or 8.0, respectively, as described above. To obtain the final stock solution that was subsequently used in the equilibrium adsorption experiments, a preliminary lysozyme solution and a salt solution of the same pH value were mixed in a 1:1 (v/v) ratio yielding ionic strengths of the studied salts varying between 0 mM and 3000 mM.

Equilibrium adsorption measurements were carried out with 50.9 μL resin and 500 μL lysozyme solution. All adsorption experiments were performed at a constant temperature of 25℃ and the equilibration time was always 6 h. UV absorption measurements at 280 nm were used for analyzing the lysozyme concentration in the liquid phase after equilibration. Lysozyme‐free solutions were measured as blank values for each studied salt, each studied ionic strength, and each studied pH value. For these batch adsorption experiments, a relative error of the equilibrium loading of the resin of about 10%, except for very small loadings, was determined by Werner et al. [43].

The experimental data of the individual isotherms were correlated with the semi‐empirical approach of Oberholzer and Lenhoff [44, 45]:

(1) $$c_{\mathrm{Lys}}=\frac{q_{\mathrm{Lys}}}{K^{\mathrm{ads}}}\;\;\cdot \exp\left[\beta \cdot \sqrt{q_{\mathrm{Lys}}/q_{0}}\cdot \exp\frac{-\gamma }{\sqrt{q_{\mathrm{Lys}}/q_{0}}}\right]$$

with *c*_Lys_ the lysozyme concentration in the liquid phase, *q*_Lys_ the lysozyme loading of the resin, and *K* ^ads^ the adsorption equilibrium constant. The lumped fitting parameters *β* and *γ* were made dimensionless by introducing *q*_0 _= 1 mg/mL. In this work, Equation (1) was used as a correlation tool only since the respective parameters (except for *K* ^ads^) do not exhibit any coherence with any of the studied process parameters (pH value, ionic strength, salt type) as, e.g., shown in Tables S1–S3. Hence, any mathematical correlation yielding a good description of the experimental data could have been used instead of Equation (1) without significantly affecting the results of this work.

### 2.3 Data processing

MATLAB was used for all data processing and modeling steps. In the following, the results are discussed in terms of the ionic strength *I*, which enables a better comparability between the four studied salts (NaCl, Na_2_SO_4_, NH_4_Cl, (NH_4_)_2_SO_4_). The ionic strength is defined based on the ion molarities here; the small concentration of buffer salts (25 mM) that was present in all solutions was thereby not considered for calculating the ionic strength throughout this work.

For extending the model from our previous work [36] to additionally describe the dependence of the adsorption of lysozyme on the pH value, the experimental data at pH 5.0 and 6.0, that were measured in this work, were used together with the experimental data at pH 7.0, obtained in our previous work [36]. The experimental data from several adsorption isotherm measurements at pH 8.0, which were additionally carried out in this work, were used for testing the predictive capability of the model for the extrapolation to further pH values only; these data were not used for developing and fitting the model and are therefore not discussed in detail in the following sections.

### 2.4 Model of the influence of salts

As in our previous work [36], an exponential correlation function was used here for describing the dependence of the lysozyme loading *q*_Lys_ of the mixed‐mode resin at a constant lysozyme concentration in the liquid phase *c*_Lys_ on the ionic strength *I* for each salt *S*:

(2) $$\begin{array}{c} \begin{array}{c} \frac{q_{\mathrm{Lys}}\left(c_{\mathrm{Lys}}=\mathrm{const}.\right)}{\mathrm{mM}}=k_{0}\;-k_{F}+k_{F}\cdot \exp\left(-k_{S}\cdot \frac{I}{1000\;\text{mM}}\right) \end{array} \end{array}$$

where *k*_0_, *k*_F_, and *k_S_* are model parameters that depend on the lysozyme concentration in the liquid phase *c*_Lys_ as described in the following.

If no additional salt is present in solution, i.e., *I *= 0 mM, *q*_Lys_ equals *k*_0_, which in turn depends on *c*_Lys_ and corresponds to the equilibrium adsorption isotherm of lysozyme in pure buffer solution. The semi‐empirical correlation function of Oberholzer and Lenhoff, which was already used for the correlation of the individual adsorption isotherms, cf. Equation (1), was applied for describing the dependence of *k*_0_ on *c*_Lys_:

(3) $$\frac{c_{\mathrm{Lys}}}{\mathrm{mM}}=\frac{k_{0}}{a_{0}^{\left(1\right)}}\;\cdot \exp\left[a_{0}^{\left(2\right)}\cdot \sqrt{k_{0}}\cdot \exp\frac{-a_{0}^{\left(3\right)}}{\sqrt{k_{0}}}\right]$$

where $a_{0}^{\left(1\right)}$, $a_{0}^{\left(2\right)}$, and $a_{0}^{\left(3\right)}$ are dimensionless model parameters that correspond to the Lenhoff parameters *K* ^ads^, *β*, and *γ*, respectively.

At very high ionic strengths, the lysozyme loading *q*_Lys_ approaches the difference *k*_0_−*k*_F_, cf. Equation (2). Similar to our previous work [36], a linear correlation function was deployed for describing the dependence of *k*_0_−*k*_F_ on *c*_Lys_:

(4) $$k_{0}-\;k_{F}=a_{F}^{\left(1\right)}\;\cdot \frac{c_{\mathrm{Lys}}}{\mathrm{mM}}$$

where $a_{F}^{\left(1\right)}$ equals the adsorption equilibrium constant of a linear adsorption isotherm, which is assumed here for very high ionic strengths.

For the salt‐specific parameter *k_S_*, which describes how strong a specific salt *S* affects the adsorption of lysozyme on the mixed‐mode resin, a correlation function similar to Equation (2) was introduced in analogy to our previous work [36]:

(5) $$k_{S}=a_{S}^{\left(1\right)}\;-a_{S}^{\left(2\right)}+a_{S}^{\left(2\right)}\cdot \exp\left(-a_{S}^{\left(3\right)}\cdot \frac{c_{\mathrm{Lys}}}{\mathrm{mM}}\right)$$

where $a_{S}^{\left(1\right)}$, $a_{S}^{\left(2\right)}$, and $a_{S}^{\left(3\right)}$ are salt‐specific model parameters.

The parameters $a_{0}^{\left(j\right)}$, $a_{F}^{\left(1\right)}$, and $a_{S}^{\left(j\right)}\;\left(j\;=\;\{1,2,3\}\right)$ depend on the pH value and were determined as described above for pH 5.0 and 6.0 from the experimental data of this work, and for pH 7.0 from the experimental data of our previous work [36].

A detailed description of the model development for the influence of the ionic strength of different salts on the adsorption of lysozyme on the mixed‐mode resin at constant pH value is given in our previous work [36]. The extension of the model to also consider the dependence on the pH value is described in detail in the results section of the present work.

## 3 RESULTS AND DISCUSSION

### 3.1 Experimental adsorption isotherms

**FIGURE 1:**
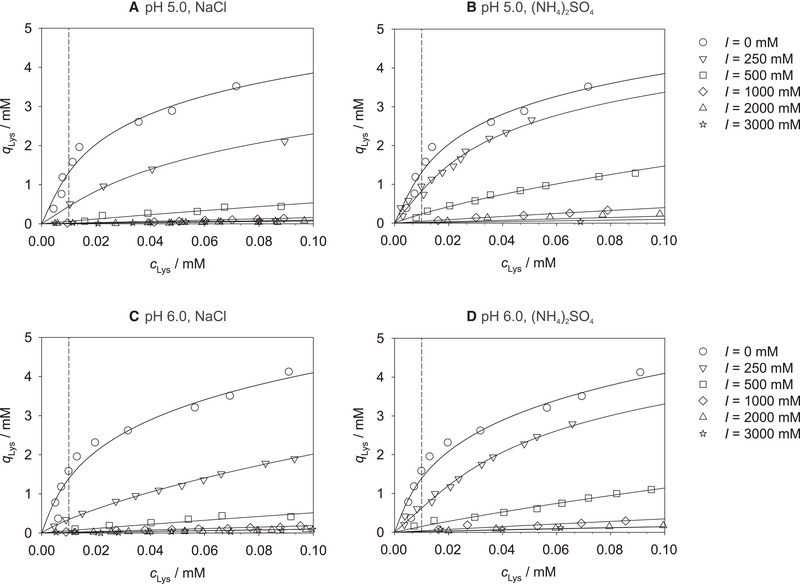
Experimental equilibrium adsorption isotherms (symbols) of lysozyme on Toyopearl MX‐Trp‐650M at pH 5.0 (A, B) and pH 6.0 (C, D) at 25℃ for different ionic strengths *I* of sodium chloride (A, C) and ammonium sulfate (B, D), and corresponding individual correlations (lines), cf. Equation (1)

Figure 1 shows the experimental equilibrium adsorption data of lysozyme on Toyopearl MX‐Trp‐650M at pH 5.0 and 6.0 for solutions containing sodium chloride or ammonium sulfate together with the corresponding individual correlations, cf. Equation (1). The remainder of the experimental data obtained in this work at pH 5.0 and 6.0, i.e., for solutions containing sodium sulfate or ammonium chloride, are shown in Figure S1. The Lenhoff parameters obtained by individually fitting the experimental adsorption isotherms at pH 5.0 and 6.0 with Equation (1) are summarized in Tables S1 and S2.

The tryptophan ligand of Toyopearl MX‐Trp‐650M thereby enables ionic interactions of lysozyme with the resin via its carboxyl group (acting as a weak cation exchanger) as well as hydrophobic interactions via its indole group (acting like a HIC resin) and therefore an interplay of the different interaction mechanisms. As already observed at pH 7.0 in our previous work [36], also the results at pH 5.0 and 6.0 of this work show significantly higher lysozyme loadings in the cation exchange region (at lower ionic strengths) than in the hydrophobic interaction region (at higher ionic strengths) for all studied salts. The highest lysozyme loadings were observed at *I *= 0 mM, i.e., when no additional salt was added to the 25 mM buffer, and the addition of salts led to a decrease of the lysozyme loading with increasing ionic strength up to approx. 1000 mM. For all studied pH values, the highest loadings for a defined ionic strength were observed for ammonium sulfate, the lowest for sodium chloride in this region. This fits well with the expectations: at low ionic strengths, the influence of the salt type is dominated by the size of the constituent ions with smaller ions (sodium and chloride ions) leading to an increased shielding effect which hampers the ion exchange adsorption. At the same time, at low ionic strengths, one could expect a stronger salting‐out effect of the chaotropic sodium chloride, i.e., an increased adsorption, compared to the kosmotropic ammonium sulfate on the HIC ligands of the resin following the so‐called reversed Hofmeister series as reported in the literature [25, 26, 27]. The results of this work indicate that for the mixed‐mode resin studied here at low ionic strengths, the different shielding effects of the salts outweigh the effects described by the reversed Hofmeister series. At higher ionic strengths, no significant dependence of the loading on neither the ionic strength nor the type of salt was found, which might be due to the in general very small loadings in this region. The influence of the pH value on the adsorption depends on the ionic strength and is therefore difficult to perceive based on the depiction in Figure 1; to facilitate studying the influence of the pH value on the lysozyme loading, an alternative representation, which includes a data reduction as described in the following section, is chosen.

### 3.2 Modeling the dependence on the ionic strength

**FIGURE 2:**
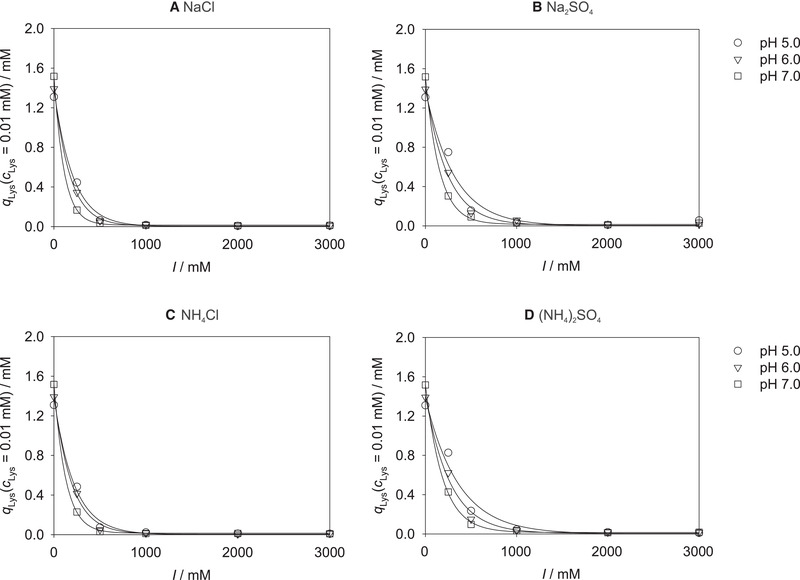
Lysozyme loading *q*_Lys_ of Toyopearl MX‐Trp‐650M at *c*_Lys _= 0.01 mM, pH 5.0, 6.0 (both this work), and 7.0 [36], and 25℃ for the four studied salts as a function of ionic strength *I*. Symbols: experimental results (correlated with Equation (1)). Lines: correlations with Equation (2)

The adsorption isotherm data at pH 5.0 and 6.0 were reduced in the same way as the data at pH 7.0 in our previous work [36]: the lysozyme loading *q*_Lys_ of the mixed‐mode resin, as given by the individual correlations, cf. Equation (1), was considered at a constant lysozyme concentration *c*_Lys_ in the interval *c*_Lys _= (0.01–0.1) mM with a step size of 0.01 mM and additionally at the very small lysozyme concentration *c*_Lys _= 0.001 mM for all studied ionic strengths. As an example, Figure 2 illustrates the results of the loading *q*_Lys_ for *c*_Lys _= 0.01 mM (cf. the vertical dashed lines in Figures 1, S1, S2, and S4) as a function of the ionic strength *I* at pH 5.0, 6.0, and 7.0 for all studied salts.

Figure 2 offers some interesting insights. Overall, the strong decrease of the lysozyme loading in the cation exchange region, which we already observed in our previous work [36], can be found for all studied pH values. The shielding of the negatively charged groups of the resin as well as of the positively charged sites on the lysozyme by the ions in solution increases with increasing ionic strength, and thereby reduces the adsorption by the cation exchange mechanism. The effect of adding a certain amount of salt is apparently directly proportional to the amount of salt already present in the solution, leading to the observed exponential decay of the loading with the ionic strength. At a defined ionic strength, the highest loadings were measured for the addition of ammonium sulfate and the lowest for sodium chloride for all studied pH values, which might be explained by differing shielding effects of the different salts due to the size of the respective ions. In the hydrophobic interaction region, i.e., at very high ionic strengths above approx. 1000 mM, adsorption was found to be independent of the ionic strength for all pH values.

In Figure 2, also the influence of the pH value on the adsorption of lysozyme can be recognized, which depends on the ionic strength. Let us first consider the behavior in solutions to which no salt was added, i.e., the data at *I *= 0 mM. A rather small but still significant influence of the pH value can be observed here with the highest lysozyme loadings at pH 7.0 and the lowest at pH 5.0, cf. also the comparison of the adsorption isotherms at *I *= 0 mM for the different pH values displayed in Figure S2A. At these conditions (only very small ion concentrations from the buffer salts are present in solution), basically no adsorption on the hydrophobic ligands of the mixed‐mode resin, but only on the cation exchange part can be expected. Furthermore, shielding effects from the few present ions can largely be neglected here. Hence, in this region, the adsorption of lysozyme is mainly influenced by the pH‐dependent net charge of lysozyme, which should lead to an increased adsorption with increasing net charge but which also amplifies the repulsion between lysozyme molecules – including that between adsorbed and non‐adsorbed molecules – and therefore can also hamper the adsorption, as well as the pH‐dependent degree of dissociation of the acid cation exchange ligands of the mixed‐mode resin. The isoelectric point (IEP) of lysozyme in aqueous solutions is at approx. pH 11.2 [46]. Thus, the net charge of lysozyme is positive at pH 5.0, 6.0, and 7.0 throughout, but its magnitude increases with decreasing pH value [46], i.e., the lower the pH value, the more amino acid residues of lysozyme carry positive charges and the more pronounced attractions between lysozyme molecules and the ionic ligands of the resin can arise. However, since at the same time the electrostatic repulsion between lysozyme molecules increases with increasing net charge, also the charge‐based exclusion of lysozyme increases with decreasing pH values, as, e.g., reported for different monoclonal antibodies [47]. Lysozyme molecules already adsorbed on the mixed‐mode resin thereby more and more impede the adsorption of further lysozyme molecules on the resin due to charge repulsion. Moreover, the dissociation equilibrium of the weak cation exchange ligands also depends on the pH value, with a decreasing degree of dissociation with decreasing pH values. A higher degree of dissociation implies that more negatively charged ligands are available for the adsorption of the positively charged lysozyme. Hence, variations of the pH value lead to opposing effects, and can therefore potentially enhance or impair the adsorption of lysozyme. At *I *= 0 mM, the influence of the electrostatic repulsion and the shifting dissociation equilibrium of the cation exchange ligands of the resin with varying pH value are apparently dominating, leading to lower adsorption at lower pH values. Results in [47] support our observations about the influence of the pH value on the adsorption on ion exchange ligands at *I *= 0 mM. This trend at *I *= 0 mM is also confirmed by the adsorption isotherms at pH 4.0 and pH 8.0, which were additionally recorded in the present work as shown in Figure S3.

Let us now consider the influence of the pH value on the adsorption of lysozyme at low but non‐zero ionic strengths up to approx. *I *= 1000 mM. The behavior at these conditions is opposite to what is observed at *I *= 0 mM: the highest lysozyme loadings are observed at pH 5.0, the lowest at pH 7.0 for all salts and ionic strengths (up to *I *= 1000 mM) here, cf. also Figure S4, where a comparison of the adsorption isotherms at pH 5.0, 6.0, and 7.0 is depicted at a constant ionic strength of 250 mM for all studied salts as example. The adsorption of lysozyme on the cation exchange part of the resin can be still expected to be dominant at rather low ionic strengths. This adsorption is, as described above, influenced by the pH dependence of the net charge of lysozyme and of the degree of dissociation of the acid cation exchange ligands of the resin and, additionally, by the shielding effects from the ions present in solutions here. The ions thereby shield both, the charges of the ligands and of the lysozyme molecules. The reversed influence of the pH value if salts are present in the solution (decreasing loading with increasing pH) compared to the absence of salts (increasing loading with increasing pH) might be attributed to a stronger shielding of the ligands’ than of the proteins’ charges as well as to a lower influence of electrostatic protein repulsion due to the shielding effects of the ions present in solution.

Let us finally consider the influence of the pH value on the adsorption of lysozyme at high ionic strengths, i.e., *I *> 1000 mM. The experimental results show weak protein adsorption in this region and a rather small influence of the pH value on the lysozyme loading for all studied salts, cf. also Figure S2B, where a comparison of the adsorption isotherms for the studied pH values is shown at a constant ionic strength of 3000 mM of sodium chloride as example.

Overall, good correlations with the introduced exponential dependence in Equation (2) were found for all studied pH values, ionic strengths, and salts, as represented by the lines in Figure 2. At this point, the modeling is done individually for each pH value, each salt, and each lysozyme concentration. In the following sections, the model is extended to also consider the dependence of the lysozyme loading on these parameters.

### 3.3 Modeling the dependence on *c*_Lys_

As described in Section 3.2, Equation (2) was fitted individually for different lysozyme concentrations in the liquid phase *c*_Lys_ and for each pH value. Hence, the parameters of Equation (2) depend on *c*_Lys_ and the pH value. The dependence on *c*_Lys_ was modeled as in our previous work [36], cf. Equations (3)–(5) in Section 2.4.

**FIGURE 3:**
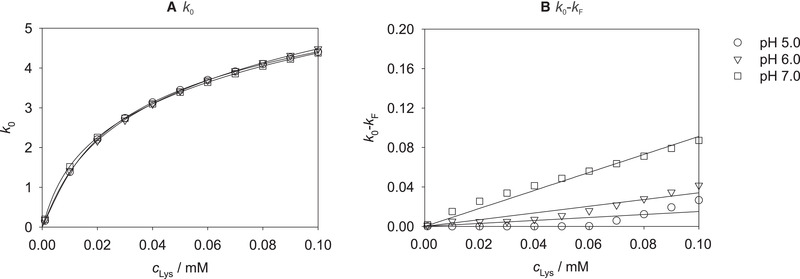
Results for *k*_0_ (A) and *k*_0_‐*k*_F_ (B) obtained by fitting Equation (2) to the experimental data plotted over the lysozyme concentration *c*_Lys_ at pH 5.0, 6.0, and 7.0 for all studied salts. Symbols: experimental results (correlated with Equation (2)). Lines: correlations with Equation (3) (A) or Equation (4) (B)

Figure 3 shows the results for the dependence of *k*_0_, which describes the adsorption isotherm at *I *= 0 mM, and the difference *k*_0_‐*k*_F_, which describes an adsorption isotherm at very large ionic strengths, on *c*_Lys_ as well as the fits with Equations (3) or (4), respectively, at pH 5.0, 6.0, and 7.0.

Figure 3A again shows the rather small influence of the pH value on the adsorption isotherms at *I *= 0 mM, which is represented by *k*_0_(*c*_Lys_). If *k*_0_‐*k*_F_ is studied as a function of *c*_Lys_, cf. Figure 3B, a slight influence of the pH value (consider the magnitude of the numerical values compared to Figure 3A) on the adsorption of lysozyme at very high ionic strengths with the highest loadings at pH 7.0 and the lowest at pH 5.0 can be observed. At high ionic strengths, basically no adsorption on the cation exchange part of the resin but only on the hydrophobic ligands can be expected as the cation exchange ligands are completely occupied with salt ions. The adsorption of lysozyme is influenced by the electrostatic repulsion between the lysozyme molecules which in turn depends on the net charge of lysozyme. An increased pH value allows a closer packaging on the surface of the resin due to the lower net charge of lysozyme leading to enhanced hydrophobic interactions and thus increasing adsorption with increasing pH value in the hydrophobic interaction region. The influence of the pH value at very high ionic strengths corresponds well with the findings for the influence of the pH value on the loading of single‐mode HIC resins. For example, Lienqueo et al. [24] and Baumann et al. [48] both described an enhanced lysozyme binding for pH values closer to the IEP due to minimized electrostatic repulsion between the lysozyme molecules.

A good description of the parameters *k*_0_ and *k*_0_‐*k*_F_ was obtained with the correlations in Equations (3) and (4) for each pH value as shown by the lines in Figure 3, although the linearity of the small difference *k*_0_‐*k*_F_ decreases slightly with decreasing pH value. The values of the model parameters $a_{0}^{\left(1\right)}$, $a_{0}^{\left(2\right)}$, $a_{0}^{\left(3\right)}$, and $a_{F}^{\left(1\right)}$ for describing the dependence of *k*_0_ and *k*_F_, respectively, on the lysozyme concentration are given in Table S4.

**FIGURE 4:**
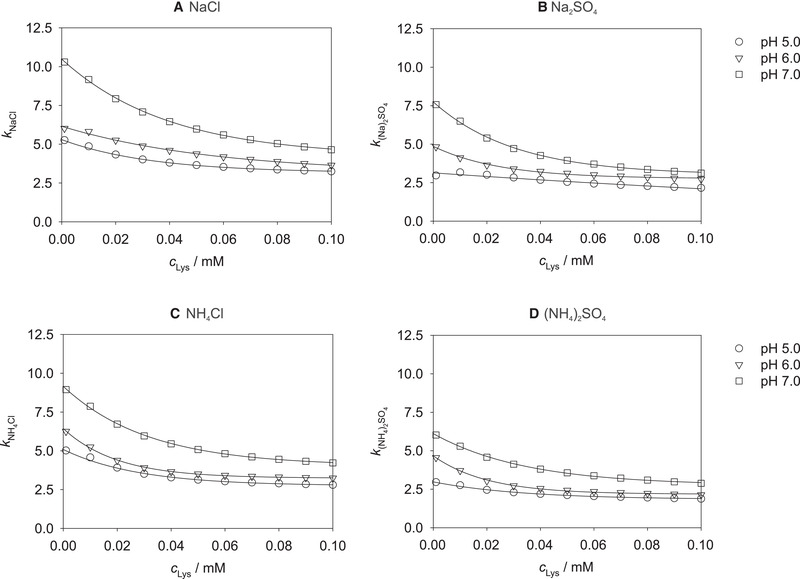
Results for *k_S_* obtained by fitting Equation (2) to the experimental data plotted over the lysozyme concentration *c*_Lys_ at pH 5.0, 6.0, and 7.0 for all studied salts. Symbols: experimental results (correlated with Equation (2)). Lines: correlations with Equation (5)

The dependence of the salt‐specific model parameter *k_S_* on the lysozyme concentration *c*_Lys_ at pH 5.0, 6.0, and 7.0 and each studied salt *S* is shown in Figure 4. *k_S_* describes how strong a specific salt *S* affects the adsorption of lysozyme on the mixed‐mode resin, cf. Equation (2): the larger the value of *k_S_*, the stronger is the impairing effect of the salt *S* at a specific lysozyme concentration and pH value.

An exponential dependence of *k_S_* on *c*_Lys_ is observed for all four salts and all pH values. Of the studied salts, the strongest influence on the adsorption, i.e., the largest *k_S_*, was found for sodium chloride, the smallest, i.e., the lowest *k_S_*, for ammonium sulfate at all studied pH values. This can be explained by a stronger shielding effect of the rather small sodium and chloride ions compared to the larger ammonium and sulfate ions.

Moreover, a significant dependence of *k_S_* on the pH value was found for all salts; the largest numbers are obtained at pH 7.0, the smallest at pH 5.0 throughout. Hence, with increasing pH value, the impairing effect of the addition of salts on the adsorption of lysozyme increases. This is in agreement with the results shown in Figure 2, in which the steepest exponential decay of the lysozyme loading with increasing ionic strength is observed at pH 7.0, which can be explained by the largest values of *k_S_* at this pH value.

Excellent correlations were obtained for all studied conditions, which are shown as the lines in Figure 4. The resulting parameters $a_{S}^{\left(1\right)}$, $a_{S}^{\left(2\right)}$, and $a_{S}^{\left(3\right)}$ of Equation (5) are summarized in Table S4. Although the dependence of the lysozyme concentration can be predicted at this point, modeling still has to be done individually for each pH value and each salt. Therefore, in the next section, the model is extended to also describe the dependence of the lysozyme loading on the pH value.

### 3.4 Modeling the dependence on the pH value

**FIGURE 5:**
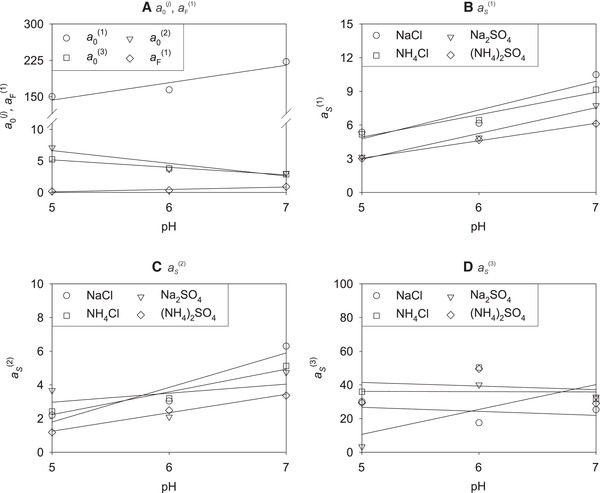
Parameters $a_{0}^{\left(j\right)}$, $a_{F}^{\left(1\right)}$, and $a_{S}^{\left(j\right)}\;\left(j\;=\;\{1,2,3\}\right)$ of Equations (3)–(5) for correlating the model parameters *k*_0_, *k*_F_, and *k_S_* on the lysozyme concentration, respectively, plotted over the pH value. Symbols: model results (correlated with Equation (3), (4), or (5)). Lines: correlations with Equation (6)

To model the dependence of the lysozyme loading on the pH value, the parameters obtained from fitting Equations (3)–(5), i.e., $a_{0}^{\left(j\right)}$, $a_{F}^{\left(1\right)}$, and $a_{S}^{\left(j\right)}\;\left(j\;=\;\{1,2,3\}\right)$, are correlated over the pH value. The results are shown in Figure 5.

Let us first consider the pH dependence of the salt‐independent parameters shown in Figure 5A. $a_{0}^{\left(1\right)}$ and $a_{F}^{\left(1\right)}$ increase with increasing pH value while $a_{0}^{\left(2\right)}$ and $a_{0}^{\left(3\right)}$ decrease with increasing pH value, cf. also Table S4. The results indicate a linear dependence of the parameters $a_{0}^{\left(1\right)}$, $a_{0}^{\left(2\right)}$, $a_{0}^{\left(3\right)}$, and $a_{F}^{\left(1\right)}$ on the pH value.

Therefore, a linear correlation function is introduced for describing the dependence of the salt‐independent parameters on the pH value:

(6) $$a_{i}^{\left(j\right)}=b_{i}^{\left(j\right)}\;+d_{i}^{\left(j\right)}\cdot \mathrm{pH}$$

where $b_{i}^{\left(j\right)}$ and $d_{i}^{\left(j\right)}$ are the pH‐dependent model parameters, which were fitted to the data for $a_{0}^{\left(1\right)}$, $a_{0}^{\left(2\right)}$, $a_{0}^{\left(3\right)}$, and $a_{F}^{\left(1\right)}$, respectively.

Let us next consider the dependence of the salt‐dependent model parameters $a_{S}^{\left(1\right)}$, $a_{S}^{\left(2\right)}$, and $a_{S}^{\left(3\right)}$ on the pH value, which is depicted in Figure 5B–D for all studied salts. The parameter $a_{S}^{\left(1\right)}$ increases with increasing pH value for all investigated salts, cf. Table S4. The parameter $a_{S}^{\left(2\right)}$ also shows a linear trend as a function of the pH value for all four salts, whereas the parameter $a_{S}^{\left(3\right)}$ does not show a specific dependence on the pH value. Nevertheless, the simple linear correlation function in Equation (6) was deployed for fitting all salt‐dependent parameters as a function of the pH value.

**FIGURE 6:**
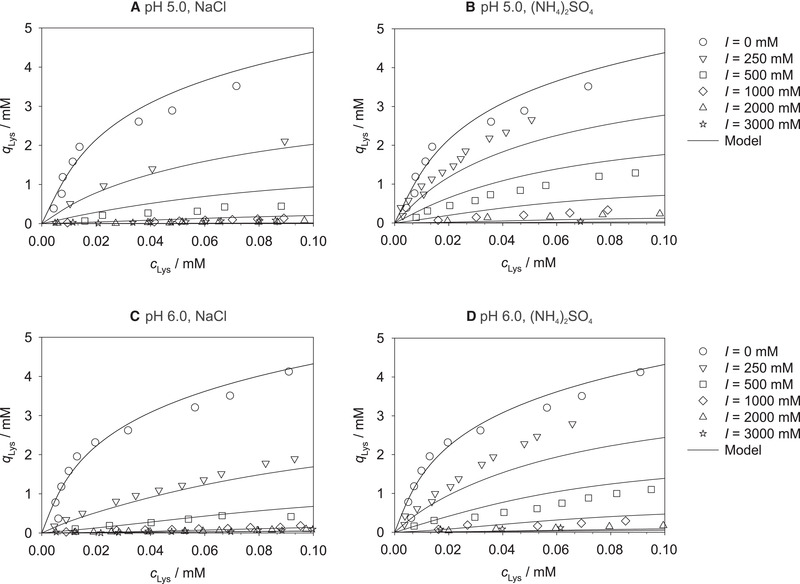
Experimental equilibrium adsorption isotherms (symbols) of lysozyme on Toyopearl MX‐Trp‐650M at pH 5.0 (A, B) and pH 6.0 (C, D) at 25℃ for different ionic strengths *I* of sodium chloride (A, C) and ammonium sulfate (B, D), and corresponding modeled equilibrium adsorption isotherms (lines)

**FIGURE 7:**
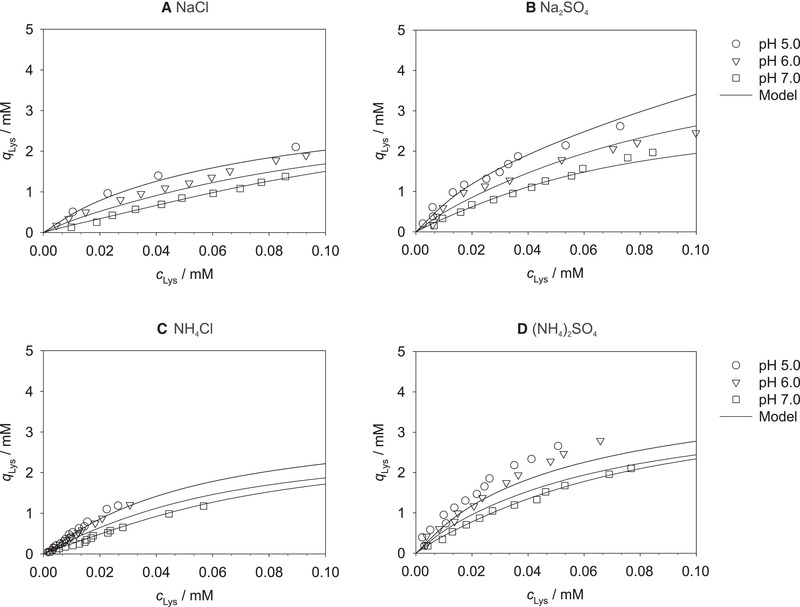
Experimental equilibrium adsorption isotherms (symbols) of lysozyme on Toyopearl MX‐Trp‐650M at pH 5.0, 6.0, and 7.0 [36] at 25℃ for *I *= 250 mM of the studied salts, and corresponding modeled equilibrium adsorption isotherms (lines)

**FIGURE 8:**
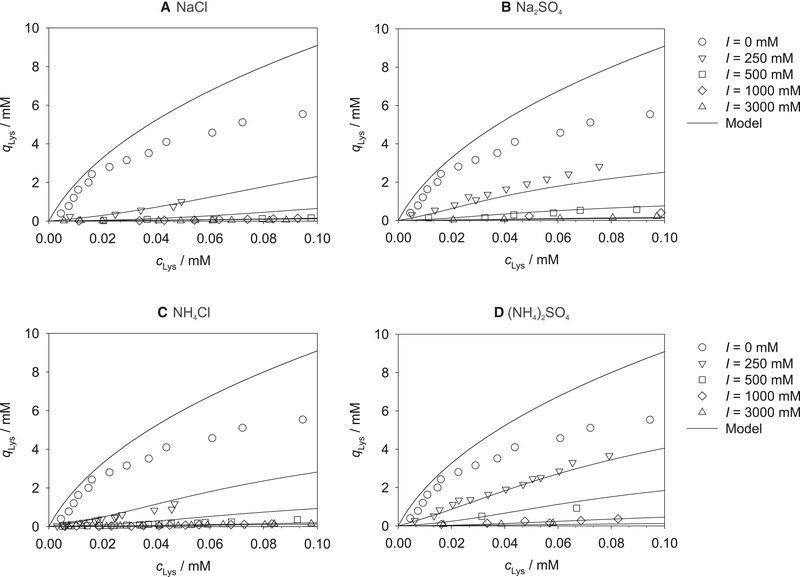
Prediction of equilibrium adsorption isotherms (lines) for lysozyme on Toyopearl MX‐Trp‐650M at pH 8.0 and 25℃ for different ionic strengths *I* of the studied salts and comparison with experimental data (symbols) of this work

As represented by the lines in Figure 5, good correlations are obtained for $a_{0}^{\left(1\right)}$, $a_{0}^{\left(2\right)}$, $a_{F}^{\left(1\right)}$, and $a_{S}^{\left(1\right)}$ with Equation (6). The correlations for $a_{S}^{\left(2\right)}$ and $a_{S}^{\left(3\right)}$ show larger deviations. However, as demonstrated in the following, these parameters seem less important for describing the lysozyme loading and therefore the approach followed here yields good predictions for equilibrium adsorption isotherms, cf. Figures 6, 7, 8 and S5–S6. The resulting model parameters $b_{i}^{\left(j\right)}$ and $d_{i}^{\left(j\right)}$, which ultimately allow modeling the lysozyme loading on Toyopearl MX‐Trp‐650M as a function of the pH value, the ionic strength of the four studied salts, and the lysozyme concentration *c*_Lys_, are summarized in Table 1.

**TABLE 1:** Parameters $b_{i}^{\left(j\right)}$ and $d_{i}^{\left(j\right)}$, cf. Equation (6), for predicting adsorption isotherms accounting for the pH value, the ionic strength of the studied salts, and the dependence on *c*_Lys_

		*j *= 1	*j* = 2	*j* = 3
$b_{i}^{\left(j\right)}$	*k*_0_	‐36.79	16.74	11.11
	*k*_F_	‐1.82	–	–
	*k*_NaCl_	‐8.05	‐8.45	38.58
	$k_{{\mathrm{Na}}_{2}{\mathrm{SO}}_{4}}$	‐8.54	0.29	‐63.17
	$k_{{\mathrm{NH}}_{4}\mathrm{Cl}}$	‐5.04	‐4.52	52.14
	$k_{{\left({\mathrm{NH}}_{4}\right)}_{2}{\mathrm{SO}}_{4}}$	‐4.72	‐4.22	36.98
$d_{i}^{\left(j\right)}$	*k*_0_	35.93	‐2.02	‐1.19
	*k*_F_	0.38	–	–
	*k*_NaCl_	2.56	2.05	‐2.38
	$k_{{\mathrm{Na}}_{2}{\mathrm{SO}}_{4}}$	2.30	0.54	14.76
	$k_{{\mathrm{NH}}_{4}\mathrm{Cl}}$	1.99	1.35	‐2.14
	$k_{{\left({\mathrm{NH}}_{4}\right)}_{2}{\mathrm{SO}}_{4}}$	1.55	1.09	‐0.17

### 3.5 Prediction of equilibrium adsorption isotherms

Inserting Equations (3)–(5) together with Equation (6) in Equation (2) yields a predictive adsorption isotherm model that includes describing the influence of the pH value, the ionic strength, and the studied salts on the lysozyme loading as a function of the lysozyme concentration in solution:

(7) $$\begin{array}{ccc} & & \frac{q_{\mathrm{Lys}}\left(c_{\mathrm{Lys}},\mathrm{pH}\right)}{\mathrm{mM}}=k_{0}\left(c_{\mathrm{Lys}},\mathrm{pH}\right)-k_{F}\left(c_{\mathrm{Lys}},\mathrm{pH}\right)\\ & & \;+\;k_{F}\left(c_{\mathrm{Lys}},\mathrm{pH}\right)\cdot \exp\left(-k_{S}\left(c_{\mathrm{Lys}},\mathrm{pH}\right)\cdot \frac{I}{1000\;\text{mM}}\right) \end{array}$$

Equation (7) can be seen as an extended version of our previously developed adsorption model in Kreusser et al. [36], which was augmented to also consider the influence of the pH value. The model enables the prediction of adsorption isotherms of lysozyme on the mixed‐mode resin Toyopearl MX‐Trp‐650M at 25℃ and pH values between 5.0 and 7.0 under the influence of sodium chloride, sodium sulfate, ammonium chloride, or ammonium sulfate for ionic strengths up to 3000 mM using the parameters given in Table 1.

To show the applicability of the model, Figure 6 shows equilibrium adsorption isotherms for solutions containing sodium chloride or ammonium sulfate as described by this model (lines) and compares them to the experimental data (symbols) at pH 5.0 and 6.0. The remainder of the model results for the experimental data of this work, i.e., for solutions containing sodium sulfate or ammonium chloride at pH 5.0 and pH 6.0 as well as for experimental data from our previous work [36], i.e., for all four studied salts at pH 7.0, are shown in Figures S5 and S6.

Excellent agreement between model and experimental data was found for all pH values, all considered ionic strengths, and all four salts. To further demonstrate that the model correctly describes the pH dependence of the lysozyme adsorption, Figure 7 exemplarily shows equilibrium adsorption isotherms for a constant ionic strength of 250 mM of sodium chloride, sodium sulfate, ammonium chloride, and ammonium sulfate for the different pH values. *I *= 250 mM was chosen since for this ionic strength, the strongest influence of the pH value was observed. The modeled isotherms (lines) are compared to the experimental data (symbols) at pH 5.0, 6.0, and 7.0 from this and our previous work [36].

Good correlations with the extended model were observed for all four salts and all pH values. The presented model correctly describes the different influence of the four salts and the pH value: the highest lysozyme loadings were obtained at pH 5.0, the lowest at pH 7.0.

The presented model can not only be used for predicting adsorption isotherms within the parameter ranges of the adsorption data recorded for developing the model, but also, to some extent, for the extrapolation to other conditions as demonstrated in Figure 8. Exemplarily, experimental adsorption isotherms of lysozyme on Toyopearl

MX‐Trp‐650M at pH 8.0 and different ionic strengths up to 3000 mM of sodium chloride, sodium sulfate, ammonium chloride, or ammonium sulfate were recorded in this work. These data were not used for developing the model or fitting the model parameters as mentioned in Section 2.3; in fact, no data at pH 8.0 was used for this purpose. However, for the sake of completeness, the Lenhoff parameters, cf. Equation (1), obtained from a fit to the corresponding experimental data of the individual adsorption isotherms at pH 8.0, are given in Table S3. These parameters were not used at any point in this work. The symbols in Figure 8 denote the experimental data at pH 8.0, the lines represent the predictions with the presented model.

For *I *>* *0 mM, the agreement of the predictions with the experimental data is excellent for all studied salts and ionic strengths. At *I *= 0 mM, a reasonable description is obtained. Overall, the results illustrate that the presented model is a valuable tool for the prediction of equilibrium adsorption isotherms of lysozyme on a mixed‐mode resin; it gives accurate predictions within the parameter ranges it was fitted to, but also yields reasonable results when used for extrapolations to other conditions that are not too far away from the training conditions, as demonstrated for the adsorption at pH 8.0.

## 4 CONCLUDING REMARKS

In the present work, we have systematically studied the influence of the pH value between pH 5.0 and 7.0 and of the ionic strength for the entire range of technically relevant ionic strengths, i.e., between 0 mM and 3000 mM, on the adsorption of lysozyme on the mixed‐mode resin Toyopearl MX‐Trp‐650M at 25℃ for four important salts, namely, sodium chloride, sodium sulfate, ammonium chloride, and ammonium sulfate. For all studied pH values and all studied salts, an exponential decay of the lysozyme loading of the resin with increasing ionic strength *I* was observed in the cation exchange region up to approx. *I *= 1000 mM, which can be explained by the shielding effect of the ions in solution. At higher ionic strengths, increasing hydrophobic interactions lead to a lysozyme adsorption that is almost independent of the ionic strength with comparably low loadings at all studied conditions. For all studied pH values and a constant ionic strength, the smallest lysozyme loadings were found for sodium chloride, the largest for ammonium sulfate, which can be explained by the more effective shielding effect of the rather small sodium and chloride ions compared to ammonium and sulfate ions, which in particular hampers the lysozyme adsorption on the cation exchange ligands of the resin. Increasing lysozyme loadings with increasing pH value were found in salt‐free solutions, which could be explained by the charge repulsion of lysozyme molecules and the shifting dissociation equilibrium of the weak cation exchange ligands of the mixed‐mode resin. In contrast, at *I *= (250–1000) mM, decreasing lysozyme loadings with increasing pH value were observed. The decreasing positive net charge of lysozyme with increasing pH value appears to be the predominant effect on the adsorption in the cation exchange region. At very high ionic strengths (*I *>* *1000 mM), higher pH values lead to higher lysozyme loadings of the resin. The adsorption in the hydrophobic interaction region can be explained by a less pronounced repulsion between hydrophobic ligands of the mixed‐mode resin and the lysozyme molecules (due to the smaller net charge) with increasing pH value.

Furthermore, we have extended a previously introduced mathematical model for describing the adsorption of lysozyme on Toyopearl MX‐Trp‐650M to also consider the influence of the pH value in this work. The model presented here successfully describes the influence of all studied pH values, ionic strengths, and salts on the lysozyme loading as a function of the lysozyme concentration in solution. Hence, the model can predict equilibrium adsorption isotherms for a wide range of technically relevant process parameters. Good agreement of the model predictions with the experimental data of this work was found. The model can even be used for extrapolations to process parameters outside the training data, which is demonstrated for adsorption isotherms at pH 8.0 after training the model to data at pH 5.0, 6.0, and 7.0 only. Ultimately, the modeling approach can be transferred to other systems, e.g., proteins, MMC resins, or salts, in a straightforward manner if sufficient training data for the respective system are available for adjusting the model parameters, and the approach can be applied for the conceptual process design and the optimization of separation processes of proteins with MMC.

## NOMENCLATURE

**Table:** 

*a*		model parameter
*b*		model parameter
*c*	[mM]	liquid phase concentration
*c* ^(^^*m*^^)^	[mg/mL]	liquid phase concentration
*d*		model parameter
*I*	[mM]	ionic strength
*k*_0_		model parameter for salt‐free solutions
*K* ^ads^		adsorption equilibrium constant
*k*_F_		model parameter for high ionic strength
*q*	[mM]	loading of the resin
*q* ^(^^*m*^^)^	[mg/mL]	loading of the resin
*q*_0_	[mM]	normalization constant
*S*		salt

## CONFLICT OF INTEREST

The authors have declared no conflict of interest.

## Supporting information



Supporting information.

## Data Availability

The data that supports the findings of this study are available in the supporting information of this article.
