# Supplementary material for: Influence of pH value and salts on the adsorption of lysozyme in mixed‐mode chromatography
Source: Eng Life Sci. 2021 Nov 8;21(11):753–68. doi: 10.1002/elsc.202100058 (PMC8576077; doi:10.1002/elsc.202100058)
Supplement: Supplementary file 1 — Supporting information. [file ELSC-21-753-s001.pdf]

Supporting Information for Influence of pH Value and Salts on the Adsorption of Lysozyme in Mixed-Mode Chromatography

Jannette Kreusser, Fabian Jirasek, Hans Hasse

Laboratory of Engineering Thermodynamics (LTD), TU Kaiserslautern, Kaiserslautern, Germany

Parameters of the Correlation of the Individual Adsorption Isotherms

The parameters of the semi-empirical correlation function of Oberholzer and Lenhoff [1, 2], cf. Eq. (1), obtained from a fit to the corresponding experimental data of the individual adsorption isotherms at pH 5.0, 6.0, and 8.0 are given in Tables S1, S2, and S3. The parameters of the correlation of the individual adsorption isotherms at pH 8.0 are shown here for the sake of completeness only. These data were not included in the model development since the additional data at pH 8.0 were measured for testing the capability of the model for the extrapolation to further pH values only. The respective parameters at pH 7.0 can be found in the Supplementary Material of our previous work Kreusser et al. [3]. Some of the parameters of the correlation of the individual adsorption isotherms correspond to either the starting or the boundary value used during fitting.

Table S1: Parameters of the correlations of the individual adsorption isotherms of lysozyme on Toyopearl MX-Trp-650M with Eq. (1) for all studied conditions at pH 5.0 and 25 ℃.

Salt	I / mM	K^{ads}	β	γ
None	0	150.10	2.13	18.22
NaCl	250	45.06	1.38	14.12
	500	6.90	0.09	0.00
	1000	1.58	0.00	10.00
	2000	0.71	0.00	10.00
	3000	0.94	0.00	10.00
Na_2SO_4	250	97.33	0.39	5.22
	500	15.59	2.48	15.94
	1000	3.69	1.73	16.31
	2000	1.06	0.02	61.08
	3000	9.31	0.57	0.00
NH_4Cl	250	48.34	12.46	29.31
	500	7.31	0.00	10.00
	1000	2.31	0.00	9.89
	2000	1.02	0.00	10.00
	3000	0.92	0.00	10.00
$(NH_4)_2SO_4$	250	84.09	3.34	22.47
	500	32.05	0.17	0.00
	1000	4.85	0.08	0.00
	2000	1.81	0.00	9.60
	3000	0.97	0.00	10.00

Table S2: Parameters of the correlations of the individual adsorption isotherms of lysozyme on Toyopearl MX-Trp-650M with Eq. (1) for all studied conditions at pH 6.0 and 25 ℃.

Salt	I / mM	K^{ads}	β	γ
None	0	185.94	0.93	11.89
NaCl	250	50.26	0.17	0.00
	500	6.33	0.07	0.00
	1000	1.87	0.00	10.00
	2000	0.98	0.00	10.00
	3000	0.96	0.00	10.00
Na_2SO_4	250	57.74	0.74	9.81
	500	15.83	0.14	0.00
	1000	7.13	0.22	0.00
	2000	1.28	0.00	10.00
	3000	1.61	4.83	1.53
NH_4Cl	250	41.56	17.72	30.71
	500	5.08	0.00	10.00
	1000	1.63	0.00	10.00
	2000	0.66	0.00	10.00
	3000	1.24	0.00	10.00
$(NH_4)_2SO_4$	250	62.47	3.52	25.03
	500	17.52	0.11	0.00
	1000	4.02	0.07	0.00
	2000	2.18	0.28	0.00
	3000	1.62	0.09	0.00

Table S3: Parameters of the correlations of the individual adsorption isotherms of lysozyme on Toyopearl MX-Trp-650M with Eq. (1) for all studied conditions at pH 8.0 and 25 ℃.

Salt	I / mM	K^{ads}	β	γ
None	0	141.78	2.78	29.57
NaCl	250	16.79	0.00	76.30
	500	2.49	0.00	10.00
	1000	1.52	0.00	10.00
	3000	0.72	0.00	10.00
Na_2SO_4	250	39.16	0.00	10.00
	500	6.28	0.01	0.00
	1000	7.01	0.31	0.00
	3000	2.22	0.25	0.00
NH_4Cl	250	21.14	0.00	10.00
	500	3.59	0.00	10.00
	1000	1.70	0.00	10.00
	3000	1.49	0.00	10.00
$(NH_4)_2SO_4$	250	50.79	0.15	16.45
	500	15.06	0.18	0.00
	1000	6.22	0.30	0.00
	3000	1.15	0.00	10.00

Additional Experimental Results

The experimental data obtained at pH 5.0 and 6.0 for solutions containing sodium sulfate or ammonium chloride are shown in Figure S1 together with the corresponding individual correlations, cf. Eq. (1).

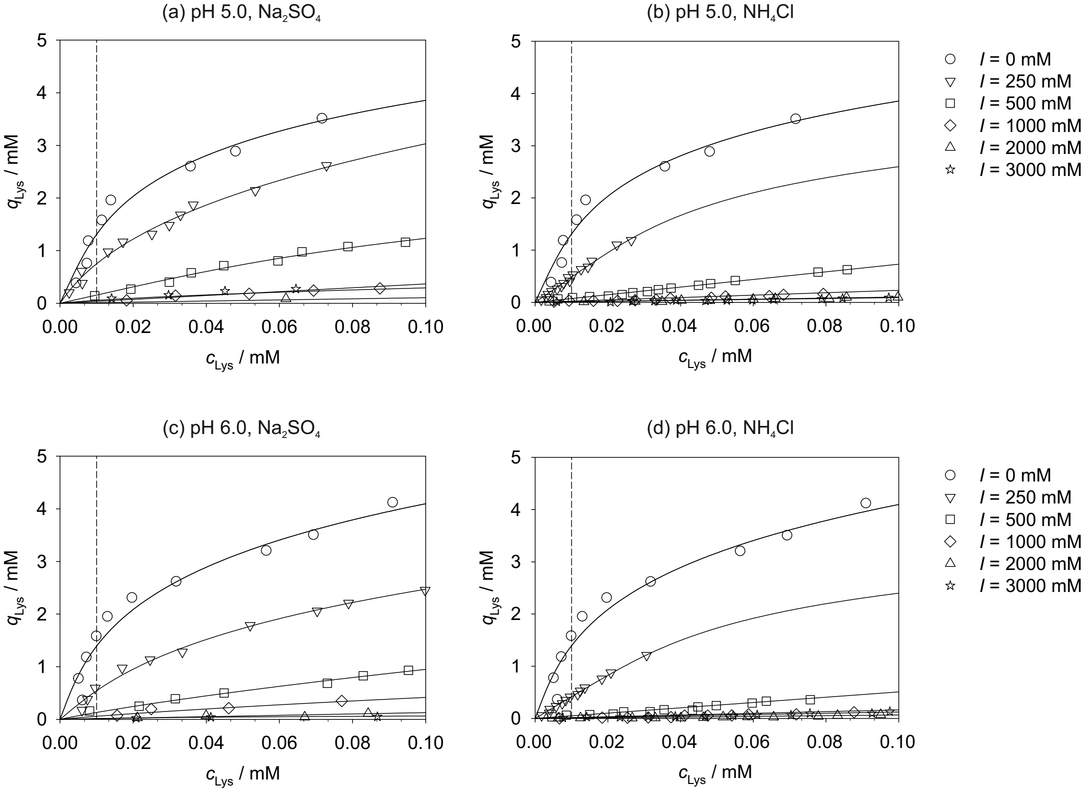

Figure S1: Experimental equilibrium adsorption isotherms (symbols) of lysozyme on Toyopearl MX-Trp-650M at pH 5.0 (a, b) and pH 6.0 (c, d) at 25 °C for different ionic strengths I of sodium sulfate (a, c) and ammonium chloride (b, d), and corresponding individual correlations (lines), cf. Eq. (1).

For both pH values and all studied salts, significantly higher lysozyme loadings were observed in the cation exchange region (at lower ionic strengths) than in the hydrophobic interaction region (at higher ionic strengths). The highest lysozyme loadings were found at $I = 0$ mM and the addition of salts led to a decrease of the lysozyme loading with increasing ionic strength up to approx. 1000 mM. At higher ionic strength, no

significant dependence of the loading on the ionic strength was found.

A comparison of the experimental adsorption isotherm data at pH 5.0, 6.0 (both this work), and 7.0 [3] without the addition of a studied salt, i.e., at $I = 0$ mM, and exemplarily at the highest studied ionic strength, i.e., $I = 3000$ mM, for sodium chloride is depicted in Figure S2 together with the corresponding individual correlations, cf. Eq. (1).

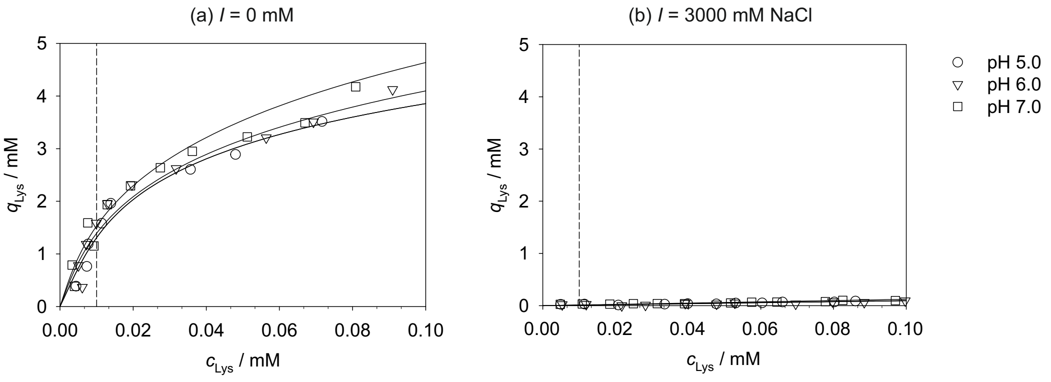

Figure S2: Experimental equilibrium adsorption isotherms (symbols) of lysozyme on Toyopearl MX-Trp-650M at pH 5.0, 6.0, and 7.0 [3] at 25 °C for $I = 0$ mM (a) and $I = 3000$ mM of sodium chloride (b), and corresponding individual correlations (lines), cf. Eq. (1).

At $I = 0$ mM, the highest lysozyme loadings were found at pH 7.0 and the lowest at pH 5.0 for all salts. Apparently, the influence of the electrostatic repulsion and the shifting dissociation equilibrium of the cation exchange ligands of the resin with varying pH value outweigh the changing net charge of lysozyme leading to an increased adsorption with increased pH value. Based on the depiction in Figure S2 (b), the influence of the pH value on the lysozyme loading is rather small at $I = 3000$ mM for all studied salts.

The lysozyme loading at $I = 0$ mM, pH 4.0 to 8.0, and $c_{\mathrm{Lys}} = 0.1$ mM is shown in Figure S3.

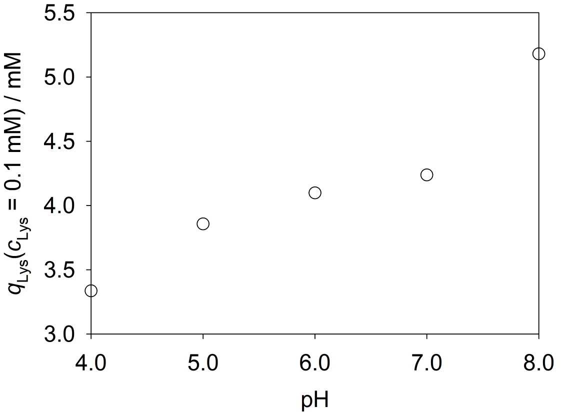

Figure S3: Experimental lysozyme loading q_{Lys} of Toyopearl MX-Trp-650M at $c_{\mathrm{Lys}} = 0.1$ mM, $I = 0$ mM, as a function of the pH value. At pH 4.0, a 25 mM sodium citrate buffer was used.

The lysozyme loading increases with increasing pH value at $I = 0$ mM; this holds for all studied pH values, namely, pH 4.0 to 8.0, and therefore supports our previous observations.

Moreover, a comparison of the experimental adsorption isotherm data at a constant ionic strength of 250 mM is displayed in Figure S4 together with the corresponding individual correlations, cf. Eq. (1) for solutions containing sodium chloride, sodium sulfate, ammonium chloride, or ammonium sulfate for the studied pH values.

By contrast, at $I = 250$ mM, the highest lysozyme loadings were found at pH 5.0 and the lowest at pH 7.0 for all salts. The reversed adsorption order compared to $I = 0$ mM might result from a stronger shielding of the ligands' than of the proteins' charges by the ions present in solution.

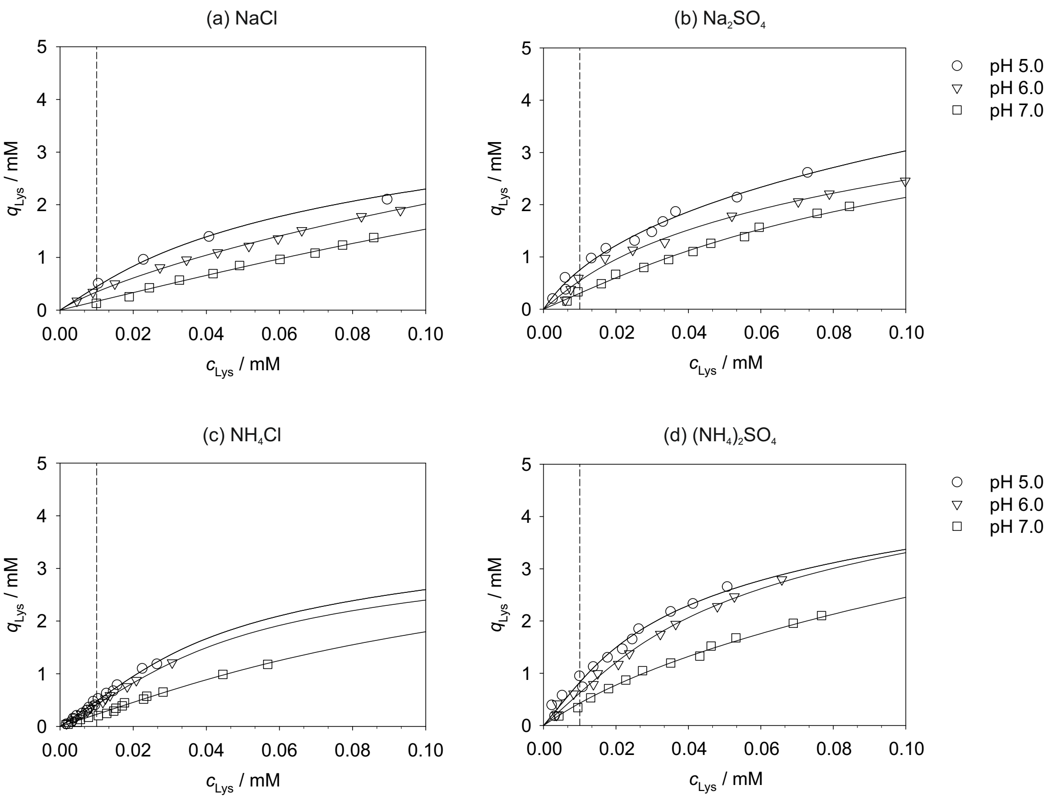

Figure S4: Experimental equilibrium adsorption isotherms (symbols) of lysozyme on Toyopearl MX-Trp-650M at pH 5.0, 6.0, and 7.0 [3] at 25 °C for $I = 250$ mM of the studied salts, and corresponding individual correlations (lines), cf. Eq. (1).

Additional Model Results

The parameters obtained for the model of salt effects in MMC at pH 5.0, 6.0, and 7.0 are given in Table S4. In contrast to our previous work [3], the experimental data at $I = 1500$ mM and $I = 2500$ mM at pH 7.0 were not included in this work for obtaining the model parameters describing the dependence of k_0, k_F, and k_S on c_Lys.

Table S4: Parameters describing the dependence of k_0, k_F, and k_S on c_Lys, cf. Eqs. (3) – (5).

pH		$a_i^{(1)}$	$a_i^{(2)}$	$a_i^{(3)}$
5.0	k_0	150.21	7.09	5.25
	k_F	0.15	-	-
	k_NaCl	5.36	2.20	30.05
	$k_\mathrm{Na_2SO_4}$	3.15	3.67	3.31
	$k_\mathrm{NH_4Cl}$	5.17	2.43	35.92
	$k_\mathrm{(NH_4)_2SO_4}$	3.01	1.18	29.37
6.0	k_0	164.09	3.69	3.80
	k_F	0.34	-	-
	k_NaCl	6.16	3.04	17.50
	$k_\mathrm{Na_2SO_4}$	4.86	2.10	40.06
	$k_\mathrm{NH_4Cl}$	6.44	3.20	50.33
	$k_\mathrm{(NH_4)_2SO_4}$	4.67	2.49	49.51
7.0	k_0	222.08	3.05	2.87
	k_F	0.91	-	-
	k_NaCl	10.49	6.30	25.29
	$k_\mathrm{Na_2SO_4}$	7.74	4.75	32.83
	$k_\mathrm{NH_4Cl}$	9.16	5.13	31.64
	$k_\mathrm{(NH_4)_2SO_4}$	6.12	3.37	29.04

The experimental equilibrium adsorption data for solutions containing sodium chloride, sodium sulfate, ammonium chloride, or ammonium sulfate at pH 5.0, 6.0, and 7.0 are shown in Figures S5 and S6 together with the corresponding isotherms obtained with the developed pH-dependent model, cf. Eq. (7).

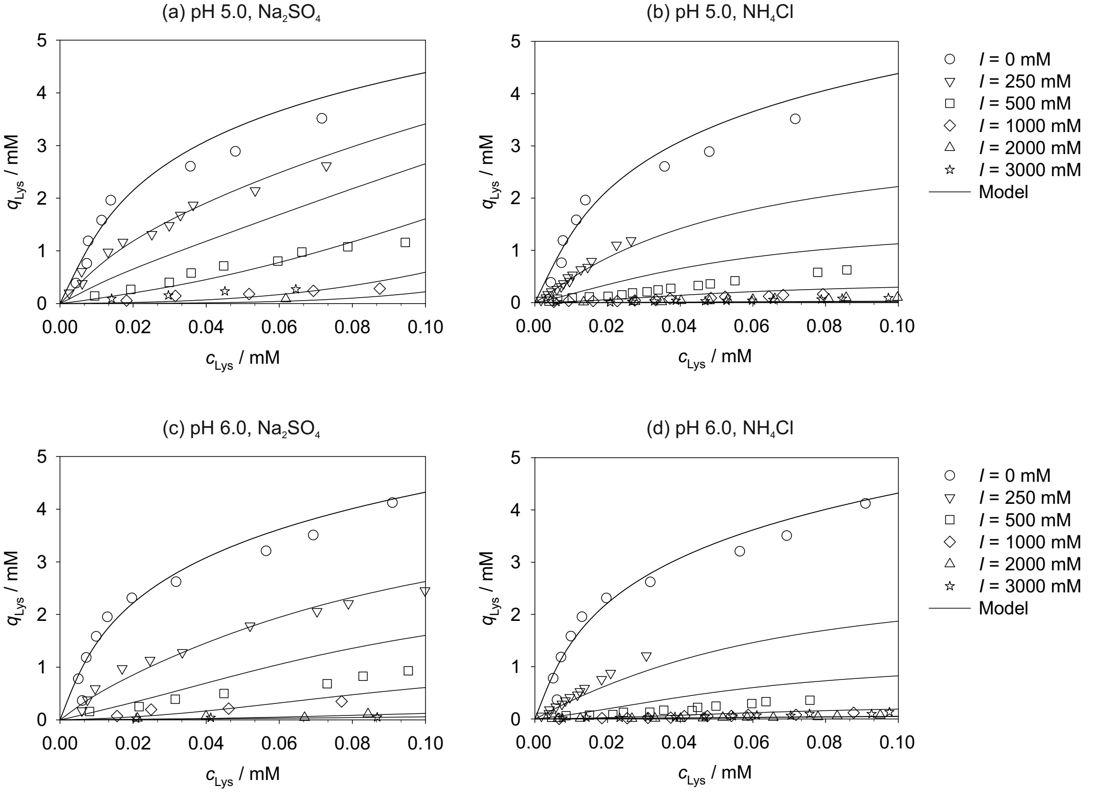

Figure S5: Experimental equilibrium adsorption isotherms (symbols) of lysozyme on Toyopearl MX-Trp-650M at pH 5.0 (a, b) and pH 6.0 (c, d) at 25 °C for different ionic strengths I of sodium sulfate (a, c) and ammonium chloride (b, d), and corresponding modeled equilibrium adsorption isotherms (lines).

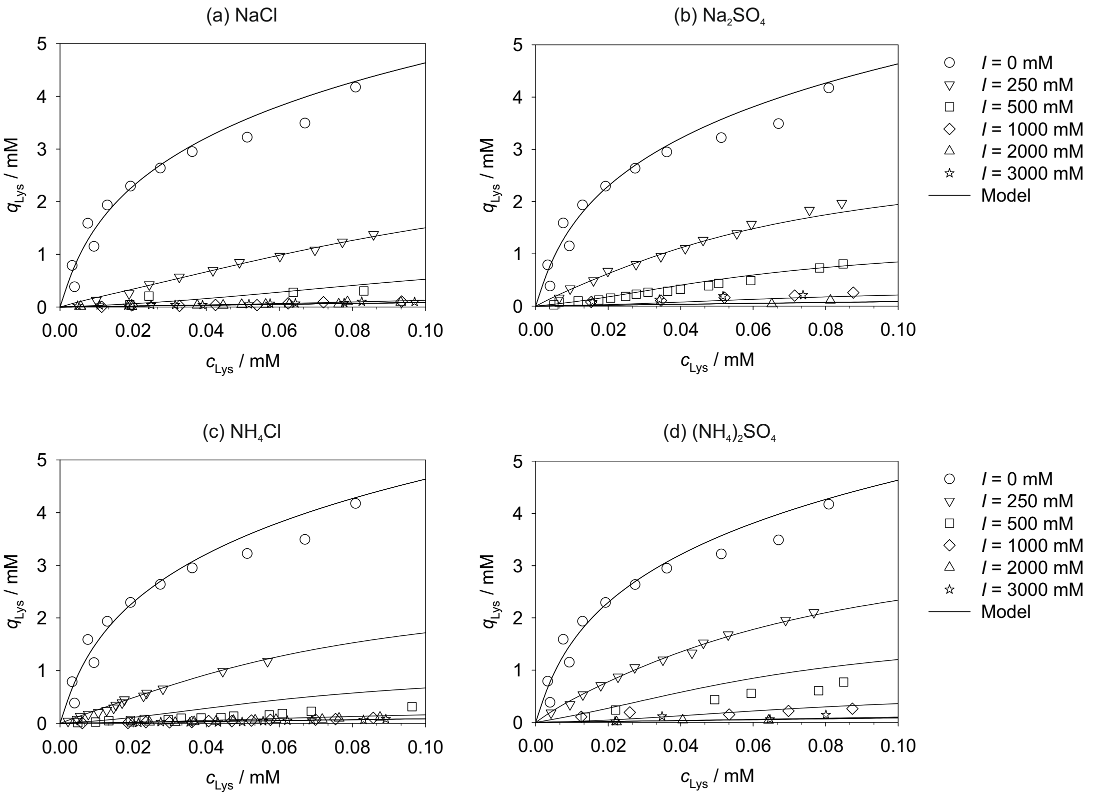

Figure S6: Experimental equilibrium adsorption isotherms (symbols) of lysozyme on Toyopearl MX-Trp-650M at pH 7.0 [3] at 25 °C for different ionic strengths I of the studied salts, and corresponding modeled equilibrium adsorption isotherms (lines).

For all studied pH values and all considered ionic strengths of the four salts, a good agreement between model and experimental data was observed. The pH-dependent model correctly describes the significantly higher lysozyme loadings at lower ionic strengths and the rather low loadings at higher ionic strengths for all salts and all pH values.

References

[1] C. Chang, A. M. Lenhoff, Comparison of protein adsorption isotherms and uptake rates in preparative cation-exchange materials, Journal of Chromatography A

827 (2) (1998) 281–293. doi:10.1016/s0021-9673(98)00796-1.

[2] M. R. Oberholzer, A. M. Lenhoff, Protein adsorption isotherms through colloidal energetics, Langmuir 15 (11) (1999) 3905–3914. doi:10.1021/la981199k.

[3] J. Kreusser, F. Jirasek, H. Hasse, Influence of salts on the adsorption of lysozyme on a mixed-mode resin, Adsorption Science & Technology 2021 (2021) 1–11. doi:10.1155/2021/6681348.
